# Decreased risk of acute myocardial infarction in stroke patients receiving acupuncture treatment: a nationwide matched retrospective cohort study

**DOI:** 10.1186/s12906-015-0828-8

**Published:** 2015-09-09

**Authors:** Sun-Fa Chuang, Chun-Chuan Shih, Chun-Chieh Yeh, Hsin-Long Lane, Chin-Chuan Tsai, Ta-Liang Chen, Jaung-Geng Lin, Tainsong Chen, Chien-Chang Liao

**Affiliations:** Department of Biomedical Engineering, National Cheng Kung University, 1 University Road, Tainan City, 701 Taiwan; School of Chinese Medicine for Post-Baccalaureate, I-Shou University, 8 Yida Road, Kaohsiung City, 824 Taiwan; Department of Surgery, China Medical University Hospital, Taichung, Taiwan; Department of Surgery, University of Illinois, Chicago, USA; Department of Chinese Medicine, E-DA Hospital, Kaohsiung, Taiwan; Department of Anesthesiology, Taipei Medical University Hospital, 252 Wuxing St., Taipei, 110 Taiwan; Health Policy Research Center, Taipei Medical University Hospital, Taipei, Taiwan; School of Medicine, Taipei Medical University, Taipei, Taiwan; School of Chinese Medicine, China Medical University, 91 Hsueh-Shih Road, Taichung, 404 Taiwan

**Keywords:** Stroke, Acute myocardial infarction (AMI), Acupuncture

## Abstract

**Background:**

Whether acupuncture protects stroke patients from acute myocardial infarction (AMI) has not been studied previously. The purpose of this study was to investigate the risk of AMI among stroke patients receiving acupuncture treatment.

**Methods:**

Taiwan’s National Health Insurance Research Database was used to conduct a retrospective cohort study of 23475 stroke patients aged 40–79 years receiving acupuncture treatment and 46950 propensity score-matched stroke patients not receiving acupuncture treatment who served as controls from 2000 to 2004. Both stroke cohorts were followed until the end of 2009 and were adjusted for immortal time to measure the incidence and adjusted hazard ratios (HRs) with 95 % confidence intervals (CIs) for new-onset AMI in multivariate Cox proportional hazard models.

**Results:**

Stroke patients who received acupuncture treatment (9.2 per 1000 person-years) exhibited a lower incidence of AMI compared with those who did not receive acupuncture treatment (10.8 per 1000 person-years), with an HR of 0.86 (95 % CI, 0.80–0.93) after adjusting for age, sex, low income, coexisting medical conditions and medications. The relationship between acupuncture treatment and AMI risk was investigated in female stroke patients (HR, 0.85; 95 % CI, 0.76–0.95), male stroke patients (HR, 0.87; 95 % CI, 0.80–0.95), patients from 50 to 59 years of age (HR, 0.75; 95 % CI, 0.63–0.90), patients from 60 to 69 years of age (HR, 0.85; 95 % CI, 0.75–0.95), patients suffering from ischemic stroke (HR, 0.87; 95 % CI, 0.79–0.95), and patients suffering from hemorrhagic stroke (HR, 0.62; 95 % CI, 0.44–0.88).

**Conclusions:**

We raised the possibility that acupuncture may be effective in lowering the risk of AMI in stroke patients aged 50–69 in this study, which was limited by a lack of information regarding stroke severity and acupuncture points. Our results suggest that prospective randomized trials are needed to establish the efficacy of acupuncture in preventing AMI.

## Background

Stroke is the second leading cause of death and a major cause of acquired disability worldwide [1]. Approximately 0.6 million people suffer from stroke in the United States annually [2]. As a major cause of long-term disability, stroke greatly influences quality of life and imposes a tremendous burden on affected individuals and their families [1]. The high risk of acute myocardial infarction (AMI) and sudden cardiac death after stroke has been documented previously [3, 4]. Patients with established coronary heart disease, peripheral vascular disease or severe stroke have a higher risk of AMI during early stroke recovery than do patients without these complications [4]. Stroke and AMI share similar atherosclerotic pathophysiologic mechanisms and risk factors, which results in a complex relationship [5].

Acupuncture, which is a complementary and alternative therapy, is the subject of growing public interest for treating stroke patients [6, 7]. The 2007 National Health Interview Survey demonstrated that 3.1 million American adults and 150,000 children had received acupuncture therapy within the past year and that approximately 1 million people began receiving acupuncture between 2002 and 2007 [8]. Acupuncture has been widely used to improve stroke patients’ motor skills, sensation, speech, and neurological functions in Asian countries [9]. Acupuncture has been used extensively as a treatment for stroke recovery in a large number of clinical studies that suggested acupuncture is effective in stroke therapy [6, 9, 10].

However, whether acupuncture is effective in preventing AMI for stroke patients remains unknown. Based on the data from the Taiwan National Health Insurance Research Database, we conducted a nationwide cohort study to investigate the risk of AMI among stroke patients receiving acupuncture treatment.

## Methods

### Source of data

Taiwan’s National Health Insurance Program, which was implemented in March 1995, covers more than 99 % of the 22.6 million residents of Taiwan. As described previously [11–15], all beneficiaries’ inpatient and outpatient medical services such as simple demographics (gender, birth date, low-income status, and urbanization of living area), primary and secondary diagnoses, procedures, prescriptions, and medical expenditures are recorded in the National Health Insurance Research Database established by the National Health Research Institutes. The research articles based on Taiwan’s National Health Insurance Research Database have been accepted in distinguished scientific journals worldwide [11–15].

### Ethics approval

The insurance reimbursement claims used in this study were from Taiwan’s National Health Insurance Research Database. To ensure privacy, the patients were de-identified in the electronic database. This study was evaluated and approved by the Institutional Review Board of Taiwan’s National Health Research Institutes (NHIRD-100-122) and E-DA Hospital, Kaohsiung, Taiwan (2014012). Informed consent was not required because the patients’ identities were decoded and scrambled. This study was conducted in accordance with the Declaration of Helsinki [11–15].

### Study design and population

We identified 182,619 new stroke survivors aged 40–79 years and older who were hospitalized (people who received inpatient care with a primary diagnosis of stroke) between January 1, 2000 and December 31, 2004 (admission date); 23475 of these patients had received at least one course (one course includes six consecutive treatments) of acupuncture after their stroke hospitalizations. To confirm that all patients with stroke in our study were incident cases, only new-onset stroke cases were included; patients with a previous medical history of stroke within five years of the index date were excluded. The diagnosis of stroke was validated as described in previous studies [11–15]. Stroke patients who received only one course of acupuncture treatment were excluded from this study. The stroke patients who did not receive acupuncture treatment were selected via matching with propensity scores (exposure vs. non-exposure ratio = 1:2). Each hospitalized stroke patient was either followed from the index date (for the non-acupuncture group: discharge date of stroke hospitalization; for the acupuncture group: the date of the first acupuncture treatment after stroke hospitalization) until 31 December 2009 or censored. For the acupuncture group, the time period between the discharge date of the stroke hospitalization and the date of the first acupuncture treatment after the stroke hospitalization was referred to as the immortal time. Therefore, immortal time bias was avoided in this study [16]. The follow-up time in person-years was calculated for each stroke patient until the diagnosis of AMI (stroke patients who visited the emergency department with a primary diagnosis) or until the patient was censored due to death or withdrawal from the insurance system or was lost to follow-up. The non-acupuncture group included patients with stroke who did not receive acupuncture treatment before the end of the follow-up period. We compared the risk of AMI between the stroke patients who did and did not receive acupuncture treatment during the follow-up period.

### Criteria and definitions

In this study, low-income patients were defined as patients who qualified for waived medical copayment as verified by the Bureau of National Health Insurance. We defined a newly diagnosed stroke according to the International Classification of Diseases, 9th Revision, Clinical Modification (ICD-9-CM 430-437). The primary outcome of AMI was defined via ICD-9-CM 410. Coexisting medical conditions were determined from medical claims for the follow-up period and included hypertension (ICD-9-CM 401-405), diabetes (ICD-9-CM 250), mental disorders (ICD-9-CM 290-319), traumatic brain injury (ICD-9-CM 800-804, 850-854), hyperlipidemia (ICD-9-CM 272.0-272.4), cardiac arrhythmia (ICD-9-CM 427.9), Parkinson’s disease (ICD-9-CM 332), depression (ICD-9-CM 296.2, 296.3), Alzheimer’s disease (ICD-9-CM 331.0), obesity (ICD-9-CM 278.0, 278.1), and malignant brain tumors (ICD-9-CM 191). Subtypes of stroke, lengths of hospital stay, admission to the intensive care unit or undergoing neurosurgery during the index stroke hospitalization were also identified as potential confounding factors in this retrospective cohort study. Cardiovascular medications such as anticoagulants, anti-platelet agents, and lipid-lowering agents were considered in this study.

### Statistical analysis

To reduce confounding effects, we developed a non-parsimonious multivariable logistic regression model to estimate a propensity score for acupuncture treatment. Clinical significance guided the initial selections of the covariates, which included age, sex, low-income status, subtypes of stroke, hypertension, diabetes, mental disorders, traumatic brain injury, hyperlipidemia, Parkinson’s disease, Alzheimer’s disease, malignant brain tumors, stays in the intensive care unit, neurosurgery, and lengths of hospital stay. We used a structured iterative approach to refine this logistic regression model to achieve a balance of covariates within the matched pairs. We then matched (without replacement) patients who received acupuncture treatment with those who did not using a greedy matching algorithm with a caliper width of 0.2 SD of the log odds of the propensity score. The nearest-neighbor algorithm was applied to construct matched pairs, assuming that the proportion of 0.95 to 1.0 was perfect. This method removed 98 % of the bias from the measured covariates [17, 18].

Chi-square tests were used to compare categorical data (i.e., age, sex, low-income status, subtype of stroke, hypertension, diabetes, mental disorders, hyperlipidemia, cardiac arrhythmia, traumatic brain injury, Parkinson’s disease, depression, Alzheimer’s disease, obesity, malignant brain tumors, smoking cessation, rehabilitation, anticoagulants, anti-platelet agents, lipid-lowering agents, stays in the intensive care unit, neurosurgery, and lengths of hospital stay) between the patients with stroke who received acupuncture treatment and the patients who did not. To compare the balance between the groups after the application of the propensity score, the means and standard deviations for age and length of hospital stay for the stroke patients who did and did not receive acupuncture treatment were compared using t-tests. We used a multivariate Cox proportional hazard model to analyze the adjusted hazard ratios (HRs) and 95 % confidence intervals (CIs) for AMI associated with acupuncture treatment in patients with stroke. All analyses were performed using Statistical Analysis Software version 9.1 (SAS Institute Inc., Cary, NC, USA). A two-sided *P* value of < 0.05 was considered significant.

## Results and discussion

After propensity-score matching (Table 1), there were no significant differences in age, sex, low-income status, subtype of stroke, hypertension, diabetes, mental disorders, hyperlipidemia, cardiac arrhythmia, traumatic brain injury, Parkinson’s disease, depression, Alzheimer’s disease, obesity, malignant brain tumors, smoking cessation, rehabilitation, anticoagulants, anti-platelet agents, lipid-lowering agents, stays in the intensive care unit, neurosurgery, and lengths of hospital stay between the stroke patients who did and did not receive acupuncture treatment.

**Table 1:** Baseline characteristics for stroke patients with or without acupuncture treatment

	Acupuncture treatment				
	No (*N* = 46950)		Yes (*N* = 23475)		*P*
Sex	n	(%)	n	(%)	1.0000
Female	20098	(42.8)	10049	(42.8)	
Male	26852	(57.2)	13426	(57.2)	
Age, years					1.0000
40–49	3994	(8.5)	1997	(8.5)	
50–59	9274	(19.8)	4637	(19.8)	
60–69	16796	(35.8)	8398	(35.8)	
70–79	16886	(36.0)	8443	(36.0)	
Low income	680	(1.5)	340	(1.5)	1.0000
Subtypes of stroke					1.0000
Hemorrhagic	4408	(9.4)	2204	(9.4)	
Ischemic	27096	(57.7)	13548	(57.7)	
Others	15446	(32.9)	7723	(32.9)	
Medical conditions					
Hypertension	35028	(74.6)	17514	(74.6)	1.0000
Diabetes	18034	(38.4)	9017	(38.4)	1.0000
Mental disorder	15722	(33.5)	7861	(33.5)	1.0000
Hyperlipidemia	6472	(13.8)	3236	(13.8)	1.0000
Cardiac arrhythmia	4492	(9.6)	2246	(9.6)	1.0000
Traumatic brain injury	3692	(7.9)	1846	(7.9)	1.0000
Parkinson’s disease	3358	(7.2)	1679	(7.2)	1.0000
Depression	664	(1.4)	332	(1.4)	1.0000
Alzheimer’s disease	24	(0.1)	12	(0.1)	1.0000
Obesity	38	(0.1)	19	(0.1)	1.0000
Malignant brain tumor	18	(0.0)	9	(0.0)	1.0000
Smoking cessation	362	(0.8)	181	(0.8)	1.0000
Rehabilitation	27350	(58.3)	13675	(58.3)	1.0000
Anticoagulant	3300	(7.0)	1650	(7.0)	1.0000
Anti-platelet agents	44612	(95.0)	22306	(95.0)	1.0000
Lipid-lowering agents	23868	(50.8)	11934	(50.8)	1.0000
ICU stay	3448	(7.3)	3448	(7.3)	1.0000
Neurosurgery	828	(1.8)	414	(1.8)	1.0000
Length of stay, mean ± SD	6.92 ± 5.15		6.94 ± 5.21		0.5655

*ICU* intensive care unit, *SD* standard deviation

During the follow-up period (Table 2), the stroke patients who received acupuncture treatment (9.2 per 1000 person-years) had a lower incidence of new-onset AMI compared with those who did not receive acupuncture treatment (10.8 per 1000 person-years), with an HR of 0.86 (95 % CI, 0.80–0.93) after adjusting for age, sex, low-income status, subtype of stroke, hypertension, diabetes, mental disorders, hyperlipidemia, cardiac arrhythmia, traumatic brain injury, Parkinson’s disease, depression, Alzheimer’s disease, obesity, malignant brain tumors, smoking cessation, rehabilitation, anticoagulants, anti-platelet agents, lipid-lowering agents, stays in the intensive care unit, neurosurgery, and lengths of hospital stay.

**Table 2:** Incidence and adjusted hazard ratios for new-onset acute myocardial infarction in stroke patients with or without acupuncture treatment stratified by age, sex, and subtypes of stroke

	No acupuncture				Acupuncture treatment					
	n	Events	Person-years	Incidence^b^	n	Events	Person-years	Incidence^b^	HR	(95 % CI)
All^a^	46950	3915	361501	10.8	23475	1376	149047	9.2	0.86	(0.80–0.93)
Sex										
Female	20098	1160	117882	9.8	10049	402	46412	8.7	0.85	(0.76–0.95)
Male	26852	1818	147873	12.3	13426	652	60251	10.8	0.87	(0.80–0.95)
Age, years										
40–49	3994	172	24672	7.0	1997	56	9388	6.0	0.84	(0.62–1.14)
50–59	9274	536	56522	9.5	4637	160	21818	7.3	0.75	(0.63–0.90)
60–69	16796	1125	97436	11.5	8398	390	38813	10.0	0.85	(0.75–0.95)
70–79	16886	1145	87126	13.1	8443	448	36643	12.2	0.93	(0.83–1.03)
Subtypes of stroke										
Hemorrhagic	4408	151	24568	6.1	2204	41	10327	4.0	0.62	(0.44–0.88)
Ischemic	27096	1847	150560	12.3	13548	671	62138	10.8	0.87	(0.79–0.95)
Others	15446	980	90628	10.8	7723	342	34199	10.0	0.89	(0.79–1.01)

*CI* confidence interval, *HR* hazard ratio. ^a^Adjusted for age, sex, low income, subtypes of stroke, hypertension, diabetes, mental disorders, hyperlipidemia, cardiac arrhythmia, traumatic brain injury, Parkinson’s disease, depression, Alzheimer’s disease, obesity, malignant brain tumors, smoking cessation, rehabilitation, anticoagulant, anti-platelet agents, lipid-lowering agents, admission to intensive care unit, neurosurgery, and lengths of hospital stay. The Akaike information criterion of the full model was 85795.051; the correlation coefficient of the Schoenfeld Residual for acupuncture and time of the full model was 0.00875, and the *p*-value was 0.5787, or no indication of lack of fit for the model. ^b^Per 1000 person-years

**Fig. 1:**
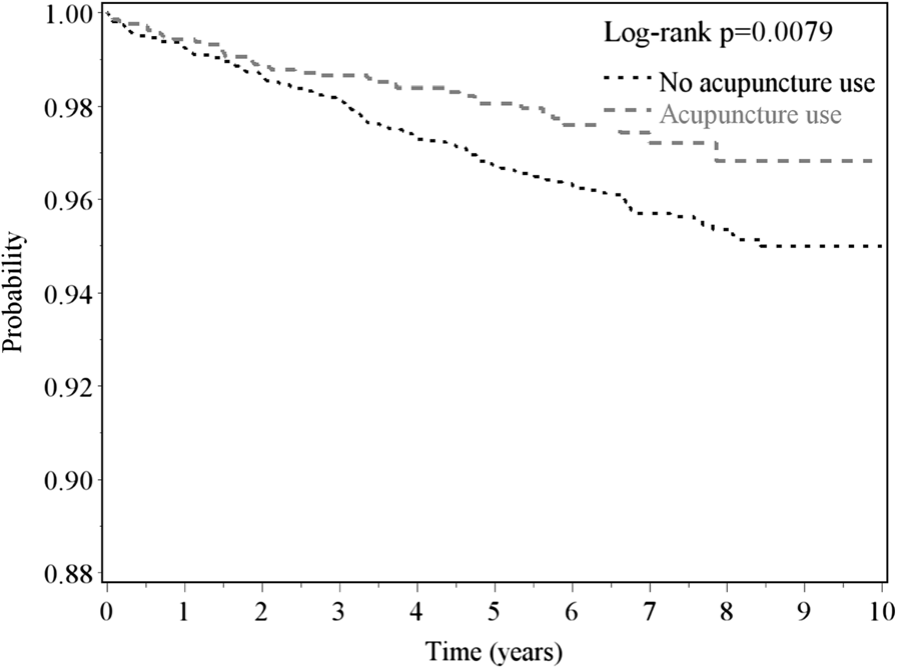
The estimated AMI-free proportions for hemorrhagic stroke patients with or without acupuncture treatment using the Kaplan-Meier method (log-rank test, *p* = 0.0079). (AMI = acute myocardial infarction)

**Fig. 2:**
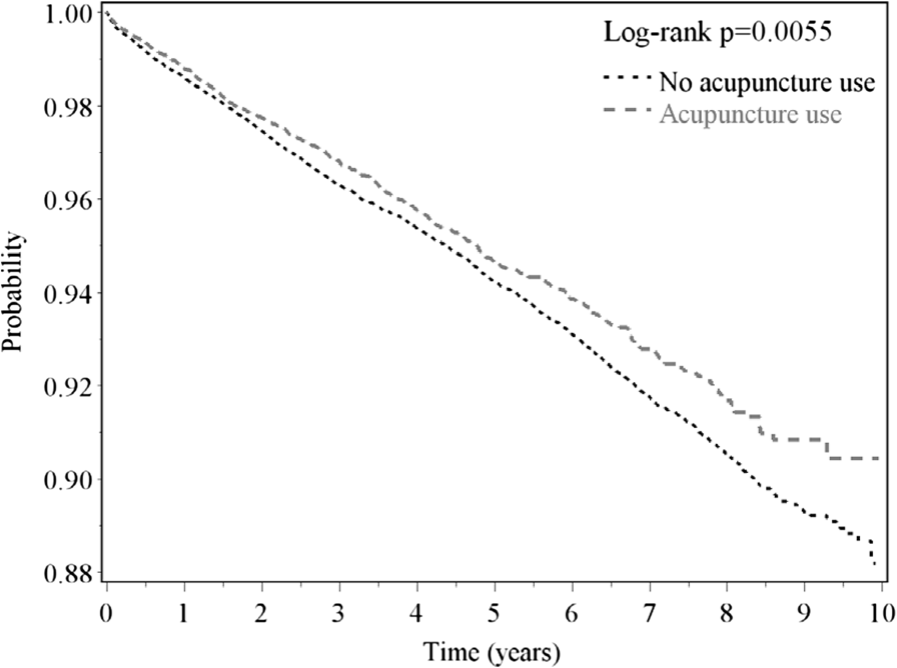
The estimated AMI-free proportions for ischemic stroke patients with or without acupuncture treatment using the Kaplan-Meier method (log-rank test, *p* = 0.0055). (AMI = acute myocardial infarction)

Among patients with stroke, the relationship between the risk of new-onset AMI and acupuncture treatment exhibited no significant difference with respect to gender (men, HR, 0.87; 95 % CI, 0.80–0.95; women, HR, 0.85; 95 % CI, 0.76–0.95). The age-stratified results demonstrated that the relationship between AMI risk and acupuncture treatment was significant in stroke patients aged 50–59 years (HR, 0.75; 95 % CI, 0.63–0.90) and 60–69 years (HR, 0.85; 95 % CI, 0.75–0.95). The beneficial effects of acupuncture treatment with respect to the reduction of AMI risk were observed in the patients with ischemic stroke (HR, 0.87; 95 % CI, 0.79–0.95) and hemorrhagic stroke (HR, 62; 95 % CI, 0.44–0.88). The log-rank test indicated that the ischemic stroke (Fig. 1, *p* = 0.0079) and hemorrhagic stroke (Fig. 2, *p* = 0.0055) patients who received acupuncture treatment had lower probabilities of new-onset AMI events than those who did not receive acupuncture treatment. In Table 3, the number of acupuncture treatments (from ≥1 to ≥15 packages) was associated with the risk of AMI in stroke patients, and the *p* value was <0.0001.

**Table 3:** Number of acupuncture treatments and risk of acute myocardial infarction in stroke patients

Acupuncture treatment	Number	Events	Person-years	Incidence^b^	HR	(95 % CI)^a^
Numbers of packages						
0	46950	2978	265756	11.2	1.00	(reference)
≥1	23475	1054	106663	9.9	0.86	(0.80–0.93)
≥2	16789	733	81701	9.0	0.79	(0.73–0.86)
≥3	13236	550	66789	8.2	0.73	(0.67–0.80)
≥4	10946	449	56537	7.9	0.71	(0.64–0.78)
≥5	9361	374	49103	7.6	0.68	(0.61–0.76)
≥6	8117	330	43027	7.7	0.69	(0.62–0.77)
≥7	7150	276	38333	7.2	0.65	(0.58–0.74)
≥8	6341	249	34381	7.2	0.66	(0.58–0.75)
≥9	5715	220	31218	7.0	0.64	(0.56–0.74)
≥10	5147	199	28312	7.0	0.64	(0.56–0.74)
≥11	4731	184	26153	7.0	0.64	(0.55–0.74)
≥12	4335	169	24206	7.0	0.64	(0.55–0.75)
≥13	3994	151	22417	6.7	0.62	(0.52–0.73)
≥14	3706	143	20854	6.9	0.63	(0.53–0.77)
≥15	3455	130	19545	6.7	0.61	(0.51–0.73)

*CI* confidence interval, *HR* hazard ratio

^a^Adjusted for age, sex, low income, subtypes of stroke, hypertension, diabetes, mental disorders, hyperlipidemia, cardiac arrhythmia, traumatic brain injury, Parkinson’s disease, depression, Alzheimer’s disease, obesity, malignant brain tumors, smoking cessation, rehabilitation, anticoagulant, anti-platelet agents, lipid-lowering agents, admission to intensive care unit, neurosurgery, and lengths of hospital stay. *P* value (Cochran-Armitage test) <0.0001

^b^Per 1000 person-years

This nationwide, comprehensive matched, immortal time-corrected cohort study reported that non-hemorrhagic stroke patients who received acupuncture treatment have a lower risk of AMI compared with those who did not receive acupuncture treatment. In particular, the effectiveness of acupuncture treatment in reducing stroke patients’ risk of AMI was observed for both sexes and patients aged 50–69 years. To the best of our knowledge, the present study was the first investigation to describe the beneficial effects of acupuncture treatment on the risk of AMI for stroke patients.

Male gender, older age, hypertension, hyperlipidemia, diabetes, traumatic brain injury, malignant brain tumors, mental disorders, Parkinson’s disease, and Alzheimer’s disease are associated with stroke [1, 2, 11, 12, 19, 20]. These sociodemographics and coexisting medical conditions are also related to the use of acupuncture [12]. To properly evaluate the relationship between acupuncture treatment and AMI risk in stroke patients, we performed a matching procedure with propensity scores to eliminate differences in sex, age, low income, age at first stroke, subtypes of stroke, ICU stay, neurosurgery, lengths of stay and coexisting medical conditions between the stroke patients who received acupuncture treatment and the stroke patients who did not receive acupuncture treatment. Multivariate Cox proportional hazard models were used to control confounding effects on the relationship between acupuncture treatment and AMI risk [21].

This study investigated the risk of AMI for male and female stroke patients receiving acupuncture treatment. In this study, the beneficial effects of acupuncture treatment with respect to the reduction of AMI risk did not differ significantly between sexes. However, this effectiveness was not observed in older patients aged 70–79 years. Older stroke patients were less likely to receive acupuncture [12], and they had more coexisting medical conditions than both young and middle-aged stroke patients [13]. Therefore, the effectiveness of acupuncture treatment with respect to the reduction of AMI risk may have been diluted by significant comorbid medical conditions [15].

### Possible explanations

To determine why acupuncture treatment has beneficial effects with respect to the reduction of AMI risk among stroke patients, we propose the following possible explanations: first, previous reports have documented that the motor ability of stroke patients improved after acupuncture treatment [6, 10, 22, 23]. Higher levels of regular physical activity and cardiorespiratory fitness are associated with a reduced risk of AMI [24]. Additionally, aerobic physical activities seem to decrease the risk of AMI [25]. Therefore, concluding that improved physical activity in stroke patients reduced the risk of AMI is reasonable. Second, an acupuncture-induced decrease in heart rate results from enhanced vagal nerve activity and decreased sympathetic nerve activity in humans [26]. Epidemiological studies have illustrated that an elevated heart rate is an independent risk factor for mortality and morbidity in patients with AMI [27, 28]. Third, both human and animal studies have demonstrated that acupuncture increases nitric oxide generation [29], a regulator of local vascular tone and blood circulation [30, 31]. A decrease in vascular nitric oxide activity likely plays a significant role in atherosclerosis development, which is an important risk factor for both AMI and stroke [30, 32]. Fourth, hyperlipidemia increases the risk of cardiovascular diseases [33, 34]. Among obese women who underwent weight loss therapy, acupuncture treatment reduced serum triglyceride, low-density lipoprotein cholesterol, and total cholesterol levels [35]. With acupuncture treatment in stroke patients, lowering lipid profiles is beneficial in reducing AMI risk.

Depression is a common, severe consequence of stroke in affected patients. Approximately 30 % of stroke patients will develop post-stroke depression, either early or late after the stroke [36]. Depression is also associated with increased risks of AMI and mortality [37]. A meta-analysis determined that acupuncture was both safe and effective in treating post-stroke depression [38]. A randomized controlled trial suggested that acupuncture significantly reduced the incidence of depression [39]. Although some of these studies had limitations (such as poor study design, inadequate adjustments for confounding factors, small samples, and inconsistent findings regarding the effects of acupuncture treatment) [38, 39] and although other studies have suggested that acupuncture has no beneficial effects on stroke outcomes [40, 41], we believe that adequate acupuncture treatment for post-stroke depression may be helpful in reducing AMI risk. Finally, patients with stroke who receive acupuncture treatment may have superior knowledge and a better attitude regarding physical rehabilitation and disease prevention, which protect against AMI [12, 42, 43].

One of this study’s limitations was that information regarding acupuncture points was not available in the National Health Insurance Research Database. The common goal of stroke patients who seek acupuncture treatment is improvement in stroke-related symptoms such as hemiplegia, dysphagia, spasticity, shoulder pain, and insomnia. However, the acupuncture treatments for stroke patients varied among different TCM physicians. We could not confirm that all TCM physicians performed the same procedures or used the same acupuncture points for the stroke patients. Usually, Taiwanese TCM physicians select acupuncture points according to textbook guidelines when performing acupuncture treatment, including points related to motor function (GB34, ST36, BL60, LR3) [44, 45], dysphagia (ST36, KI3) [46], spasticity (PC6, HT1, SP6, LU5, BL40, GB20) [47], depression (HT7, LI4, ST36, SP6, LR3) [48], and insomnia (HT7, PC6) [49].

### Study strengths

The large patient sample was one of this study’s strengths; this study used data from Taiwan’s National Health Insurance Research Database. In addition, the design of this study was based on a retrospective cohort, which provides more evidence than either case–control or cross-sectional studies. Furthermore, to remove the confounding effects of sociodemographics and coexisting medical conditions on the relationship between acupuncture treatment and AMI in stroke patients, we used a matching procedure with propensity scores to select acupuncture treatment and non-treatment controls. To control the confounding effects with respect to the relationship between acupuncture treatment and post-stroke AMI risk, multivariate Cox proportional hazard models were utilized to adjust for potential confounding factors. Finally, immortal time in observational studies may have biased the results in favor of the treatment group, but this effect is difficult to identify and avoid [50]. To reduce time-dependent bias, we calculated person-years starting from the initial acupuncture treatment to correct for immortal time in the group that received acupuncture treatment.

### Study limitations

To correctly interpret this study’s results, some limitations must be noted. First, we used retrospective medical claims data from a health insurance registry, which lacked detailed patient information regarding family history of AMI, lifestyle (such as smoking, alcohol drinking, and level of physical activity) and physical (such as body mass index, and heart rate), psychiatric, and laboratory examinations. Second, we used ICD-9-CM codes claimed by physicians for stroke without clarifying disease severity using the National Institute of Health Stroke Scale. Third, the data provided by insurance claims may have underestimated the prevalence of stroke due to cases in which patients with very minor strokes did not seek medical treatment. In addition, our study could not verify the actual acupuncture points used for treatment due to limited information from the National Health Insurance Research Database. Finally, the acupuncture treatments used for patients with stroke varied among different traditional Chinese medicine (TCM) physicians. We could not confirm that every TCM physician performed the same procedure or used the same acupuncture points for the patients with stroke.

## Conclusions

This nationwide cohort study with a comprehensive study design suggested that hemorrhagic and ischemic stroke patients who received acupuncture treatment had a lower risk of AMI compared with those who did not receive acupuncture treatment. The relationship between acupuncture treatment and the risk of AMI among stroke patients existed for both sexes and for patients aged 50–69 years; however, there was a trend toward significance in the following age categories: 40–49 and 70–79 years. The mechanism underlying the effect of acupuncture on AMI risk warrants further investigation. This study is preliminary; therefore, its results require confirmation with an appropriately designed investigation.

## Abbreviations

AMI: acute myocardial infarction
CI: confidence interval
HR: hazard ratio
ICD-9-CM: International Classification of Diseases, 9th Revision, Clinical Modification
